# Detroit Academy of Medicine

**Published:** 1871-06

**Authors:** 


### *Detroit Academy of Medicine.*

---

May 23, 1871.

Dr. McGraw read a paper on the injury caused by the indiscriminate use of alcoholic stimulants and opiates by physicians in their prescriptions. He said:

In discussing the question before us this evening, I shall take for granted, 1st, that a large part of our population are prone to the habitual use of intoxicating drugs or drinks; and 2d, that such habitual and excessive use is the direct cause of incalculable woe to many people. It is our duty, as physicians, to inquire whether we can be held responsible for any considerable portion of the misery thus produced, on account of having, by advice or prescriptions, encouraged the development of such tastes and such habits? Have desires, which might have lain dormant, been stimulated to activity by mistaken medical counsel? If so, in what patients and in what diseases? If so, how can we guard ourselves in the future from repeating the errors of the past? As regards the fact, it cannot be doubted cases of confirmed drunkenness, where liquor was first used by the advice of a physician, of confirmed opium eating, where the drug was first ordered for the relief of pain, have been met with by all physicians. How numerous such cases may be, it is difficult to determine; but it is surely a subject worthy of our most careful study, and it behooves us as physicians to inquire earnestly as to the possibility of the abuse of remedies that intoxicate, and to learn when to avoid their exhibition.

Let us, first, turn our attention to the cases of opium eating, consequent upon its medical exhibition. They will be found, I think, to be more frequent than cases of drunkenness arising from like cause. I have met, in my practice, with many cases of this description, and I cannot recall one where the habit did not begin by the use of the drug as a medicine. The histories of all such cases are strikingly similar. A patient, racked with pain, is directed to take morphine or opium. The disease becomes chronic, the pain continues, and the drug, which relieves it, is resorted to again and again. Finally, time or treatment may cure the disease, and an effort is made to discontinue the opiate, and then first the patient and his friends learn how strong the hold is which the drug has on his habits and physical ease. It is called into use again, but no longer as an anodyne, but as an intoxicant. Now, it has seemed to me that in many of the cases which I have met with, the drug had been employed by the physician to an unjustifiable extent. In many cases it has seemed to me that the patients sank into the condition of incurable opium eaters, because their medical attendants treated symptoms instead of pathological conditions; because they directed their attention to the relief of pain and *not* to the cure of the disease. This occurred from various reasons. I have seen several women who have acquired the habit of taking opiates, by employing them originally for the pain of menstruation, or for uterine colic at non-menstrual periods. I have known such women to recover afterwards from their uterine diseases, which caused their pains, and yet to find it impossible to stop the use of the familiar drug. In some of these cases no examination of the womb had ever been made, to ascertain the cause of the constantly recurring pain, but temporary relief had been given at the expense of the patient's future happiness. Now, it is by no means a pleasant task to make uterine examinations, and where the patient is an unmarried girl, every sensitive physician must feel reluctant to resort to an expedient which cannot fail to injure more or less the delicacy of a maiden. And yet, where disease exists to such a degree as to cause constantly recurring paroxysms of pain and the habitual use of opium or its compounds, I do not think that a physician does his whole duty who does not urge upon his patient and her friends the necessity of treatment. The moral injury arising from the local treatment of the womb, when conducted with scrupulous delicacy on the part of the physician, cannot begin to equal the great harm which would be experienced by a girl's moral, mental and physical nature, by the acquirement of the habit of using opium as an intoxicant.

Again, cases arise where long continued pain may cause long continued use of opiates, because the medical attendant is too timid to apply the proper treatment for the cure of the disease. I have such a case under treatment at the present time, in the person of a young woman, who has had for two years an inflammation of the periosteum of the lower third of the tibia. During that time she has suffered excruciating pain, and has acquired the habit of taking opiates. A few weeks ago I operated on her, chiseled away a bony deposit which had formed under the periosteum, and she is now well of the pain, but, alas! trying with much suffering to break off the terrible habit which constant suffering had induced. An operation, not necessarily severe, would in this case have saved great suffering, and moral and mental, as well as physical, injury.

The inherent difficulties of diagnosis of many painful diseases make it unavoidable that medical men should, in very many cases, treat the symptom, the pain, and not the disease. In many forms of neuralgia it is almost impossible to discover the real pathological condition, and all treatment is necessarily empirical, and yet it may be said, without exaggeration, that the majority of physicians are not sufficiently alive to the danger to which such treatment may expose their patients. It might be a good rule to adopt, that long continued painful diseases, whose nature is not thoroughly understood by the attending physician, should demand consultation.

In fatal diseases, like cancer, no scruples need, of course, disturb the practitioner as to the far distant effects of the remedies. In such maladies, the present relief is everything. But in obscure or slow diseases, which may be expected to end in partial or complete recovery, temporary immunity from sufferring should not be purchased at too great a cost; nor, in my opinion, is a physician justified in using opium in such cases habitually for the relief of pain, until every possible effort had been made to discover the nature of the malady and to effect its cure.

The diseases which lead to the abuse of alcoholic stimulants as a remedy, are of a different nature than those I have just been discussing. While the abuse of opium arises from its virtue in relieving pain, that of alcohol proceeds from its powers as a stimulant. I do not believe that the use of this remedy in short-lived diseases often leads to the formation of the habit of drunkenness. I believe, from my own observations, that no physician need hesitate to use brandy or whisky, to give his patient temporary support after fevers or acute inflammations. This, however, cannot be said of diseases of a chronic nature, like consumption and dyspepsia. It is a favorite practice with physicians to treat consumption by means of alcoholic stimulants. This practice is certainly not without great danger to the individual, if at all predisposed to the excessive use of alcoholic stimulants. One of the most brilliant medical men whom I have ever met suffered intensely in his struggles to throw off the habit of drinking, which he had acquired when curing himself of consumption by the use of whisky. These cases are unfortunately not rare, though not so frequent, as far as my observation extends, as those in which the habit of opium eating is thus acquired. Every circle of friends, almost, can refer to some one whom whisky first cured of consumption to kill afterwards by a more hideous death.

But of all diseases, a physician should most beware of dyspepsia, as far as regards prescribing the use of alcoholic stimulants. I call upon you, gentlemen, to remember how many old topers you have met with, who have taken to whisky, first of all, to relieve dyspepsia, and who afterwards continued its use, to moral, mental and physical destruction.

The very nature of the disease, in fact leads to self-indulgence. A dyspeptic is always a hypochondriac, and in many cases his hypochondria produces the dyspepsia. His mind constantly bent on examining the functions of the stomach, necessarily disturb that organ and derange its action. Order such a man a sufficient quantity of brandy, and he feels not only a direct stimulus to the stomach, but his spirits are raised, his blood rushes with greater vigor through his veins, and he experiences great temporary benefit. He feels that it does him good, and takes it accordingly, until it masters him completely.

In all chronic diseases, it would seem to me, except in their stages of absolute incurability, the habitual use of intoxicating drinks should be discouraged.

It would be well to remark, before closing this very imperfect paper, that great differences exist in the predisposition to drunkenness in different individuals. Born and bred in this city, and able to watch the careers of very many old acquaintances and school-fellows, I have noticed that the children of men who drank habitually, however moderately, have almost invariably turned out to be drunkards. That which is a taste in the father becomes a passion in the son. This fact should warn us especially against prescribing liquors for patients who inherit drinking tendencies.

There is also more danger of acquiring bad habits in youth than in mature age, and we may order for a middle-aged man a course of treatment which would be fraught with danger to a young one. An absolute guide, when and to whom we may prescribe remedies that may intoxicate, cannot, in my opinion, be established. While it is well for us all to recognize the dangers which may arise in the practice of our profession, we must, after all, fall back upon that good common sense, without which no man can be a good physician, for our support in all doubtful questions.

Dr. Gustin.—We, as physicians, have very little to do with the question of the abuse of these remedies. I do not prescribe them unless they are needed, and would not throw either of them out of use. It would be too great a sacrifice.

Dr. Stewart.—I was very much interested in the paper read by Dr. McGraw, and in a great measure agree with his views. If it were possible, I would do away with these agents altogether in medicine, but in the present state of our knowledge we have no other remedies to take their place. The use of alcohol and opium, as prescribed by the honest and intelligent physician of the present day, never produces harm, except in the case of an already confirmed drunkard or opium-eater, where anything which would prolong his life would lead to the same result. Their abuse is where taken as a beverage, or to excess, and we cannot deny that many of our fraternity have, both by example and precept, given strong color to the allegation laid to our charge—that we prescribe them, under all and every circumstance, in a very loose manner. A member of the Legislature told me, that should the prohibition law be enforced, any physician would prescribe all the liquor any person wished for. A liquor dealer informed me, that a certain doctor made his and the brewer's fortune by prescribing his beer ; and a certain hotel keeper has said, that by paying the doctor a percentage for prescribing it, he could sell all the liquor he wanted. This is a state of things which we, as a society, should raise our voice against. That many of us, through thoughtlessness or carelessness, may have prescribed alcohol, I admit; but that as a body, and as a rule, we do so, I deny. A trait of human nature, from Adam downwards, has been, when doing wrong, to quiet the conscience by attaching the blame to others; and who, in such cases, so handy a scape-goat as the doctor? Every person who uses alcohol or opium to excess, will say, it has been prescribed by the doctor, which may have been ten years before, and when needed. This brings to mind the evil often arising from renewing prescriptions, not only when alcohol and opium are ordered, but a great many other remedies. Quacks and charlatans take advantage of the popular idea, and dubbing themselves M. D., prescribe these remedies *ad libitum*. During the time which I spent in a drug-store, I noticed that most all the prescriptions coming from this class were roots to be put in whisky, a wine-glass full to be taken three times per day until better, which very often took a year and more. We were selling sarsaparilla in one-half gallon bottles, composed of alcohol and treacle. It is a common thing, when people are a little unwell, to have a tonic prescribed in the form of a few ounces of roots in a gallon of whisky or wine, or a keg of beer. A very old and well known prescription is, one ounce of bark to a quart of port wine, one wine-glass full three times per day. I have known a great many men to have been made drunkards in this way.

In regard to opium, it is generally known that it relieves pain. It is sold in every corner grocery and country store, with directions, and people will buy and take it on their own responsibility. Ninety-nine out of the hundred nostrums sold under the name of bitters, tonics and patent medicines in the liquid form, contain a very large percentage of alcohol, opium or some other narcotic equally injurious. These are scattered broadcast through the land, lauded by the public press as a panacea for all human ills. The names of eminent members of our profession are used without their consent, and thus the baneful traffic goes on. Is it then a wonder that so much of them should be taken, and that bad results should follow? But for the reason they are sold under the name of medicine, the public lose sight of the real culprits, and charge the honest practitioner with all the mischief done. During a long course of practice, I have never met with a case of drunkenness or opium-eating which, upon investigation, could be traced back to the prescription of a regular physician in his professional capacity.

Dr. Gilbert.—I cannot agree with Dr. McGraw that drunkenness is increased by physicians' prescriptions. The appetite is always, I believe, acquired previously, and hence I protest against this imputation cast upon the profession. I cannot shut my eyes to the fact, that some few physicians may have abused alcohol and opium by their indiscriminate use of them, but this must not be charged to the profession as a whole. But, even admit that it was true, I assert in the face of it, and defy successful contradiction, that no sound argument can be drawn from the abuse of an article that is good in itself; to do so would set aside every good gift of nature.

Now, the grounds on which it is attempted to discontinue, more or less, the use of alcohol in medicine, are that it, through a peculiar temperament, etc., may induce a species of insanity, and as a consequence, drunkenness. But this argument again, I hold, is not to the proper use of, but the abuse of it. The use of it, as a beverage, subsequent to its use as a medicine, lies not within the province of the physician to countermand. That the patient would not have used it, had it not been prescribed, cannot well be proved, neither that it necessarily *was continued because* prescribed. Drunkenness is denounced as a sin—not insanity. Insanity, I believe, is not yet discovered to be a crime. Men do not voluntarily become insane, but the crime of inebriation is so much under the control of the human will, that not to assert its independence in abstaining from it, is to foster the very crime we wish to discourage. I assert, without any hesitation, that were it morally certain that a patient would become a drunkard as the consequence of the use of alcohol necessarily administered to save his life, the physician's duty is to use it, however much he might regret the unfortunate result. The value of alcohol in relieving pain is becoming so generally known that there is a strong inducement, from its cheapness and agreeable effects, to prescribe it without consideration of the true pathology of the disease. This I regard as a great error. Indeed, it ought never be given for the *relief* of *pain;* other means are equally efficient, and are not likely to be followed by its continuance to appease a morbid appetite.

Dr. Lathrop.—Prof. Gross used to say that it was none of our business what habits our patients acquired by the proper administration of drugs (but afterwards modified his views), and the profession generally admit this. If we admit that we are to some extent responsible, we shall be constantly trammeled in our efforts to cure our patient of disease, for fear of the after consequences. Of course we have our moral responsibilities in preventing the increase of drunkenness, the same as every one else.

Dr. Book.—Physicians are blamed for much they are not accountable for. Alcohol is found everywhere, and patients acquire their bad habits before they come under our care. I had a patient some time ago that had a severe attack of pleurisy, complicated with pneumonia. His expectoration was enormous, and he was rapidly sinking. Dr. Kane was called in consultation, and we agreed upon the free administration of brandy. We hesitated some, for before his sickness he had spreed it some; but knowing that something had to be done, and that immediately, we gave the brandy. For a long time he used two bottles, holding a pint and a half, per day. He recovered, and during his convalescence we gradually withdrew the brandy, and within two weeks from the time that he was drinking two bottles per day, he had stopped the use of it altogether, and he is as temperate now as he ever was. In all the practice that I have done, I never knew a case of drunkenness or opium-eating to result from my prescriptions.

Dr. Inglis.—I endorse Dr. McGraw's paper fully, and I only regret that he did not go further. If we narrowed our use of alcohol down to the case that Dr. Gilbert has given, where it was alcohol with life and drunkenness, or its absence and death, we would have to run all risks and give it. The doctor's supposed case, however, is never actual, and I have never yet met with a case where I have had to give it to save them from dying. I think there is a lack of due sense of responsibility as to the consequences in the indiscriminate prescribing of alcohol and opium. In cases of consumption, where they will die any way, if alcohol can add in any way to their comfort, I would give it freely, for I know it could not possibly cause any bad results. I would be guided by the same feelings in regard to the free administration of morphia to a patient suffering from a cancer. Cases differ in regard to the responsibility we should feel in giving these remedies. I know of men that, like Gough, could not control their appetites if once aroused either by the administration of medicines or in partaking of the Sacrament. We should use every effort in our power to discountenance the use of alcohol, and when we are compelled to use it as a medicine, stop it as soon as possible, and urge that it be used no more. We are compelled to use opium more often than alcohol, and the same caution should be used in its administration. I never let my patients know when I prescribe opium, and it is impossible for them to acquire a habit from a remedy when they don't know what it is.

Dr. Connor.—This question we have been discussing, I consider most vital in its importance. Alcohol and opium are the sharp tools of the profession, and should be used most carefully. They are capable of doing a large amount of good or harm. The physician should settle definitely in his own mind when to use them, and only use them then. Opium destroys nerve matter by a long continued use of it, and I think alcohol acts in the same way to produce physical change, and in this way produces insanity. The longer either of these drugs are taken, the longer it takes to recover the normal condition. I never give alcohol unless I know that there is nothing that will do as well. As a profession, we aspire to a higher dignity than a mere pill-vending or dispensing of drugs. It is our duty to teach people how to live, how to obey the laws that God has established in the organism. The temperament has great influence in regard to the administration of these potent drugs. The nervous and sanguine are affected very much by their use, acquire rapidly the habit of using them, and resist strenuously all efforts to break off using them. We should go all over and through our patients, inquire into their habits, idiosyncrasies, etc.

Dr. Carstens.—It seems that physicians are too often accused of inducing bad habits that some people are subject to, with no kind of foundation. It is so with alcohol and opium. I have met, perhaps, as many opium-eaters as any member present, for while in the drug business I was called upon to sell it every day. I only remember of one case where the habit was contracted by following the physician's prescription. Everybody knows the effect of opium, and being easily obtained, they take it often, and the habit is acquired. I know of instances where ladies, not having anything to do, where, perhaps, there was a lull in the rounds of their excitement, slept after dinner to pass away time, and at night, when the proper time for sleeping came, it could only be produced by a dose of opium, and this continuing time after time, the habit was formed. It is astonishing what quantities some people will take. I knew a lady who would eat up one drachm of morphia every four days, or fifteen grains per day. It would be an interesting subject for discussion how to prevent the spread of this malady. I am sure if people could not so readily obtain this drug, very few would fall into the habit of eating it. By alcohol, I understand a compound containing the compound radical $C_4$ $H_6$ $O_2$, but if we prescribe it we must consider that wine contains carbonic and gallic acid, sugar and sulphates ; beer holds the active principle of hops in solution ; whisky contains that poisonous of all oils, fusil oil, while in gin the diuretic properties of juniper berries are found. Prescribing these remedies, we must consider the modifying influences of these adjuncts. However, when either of these are taken for a long time, the action of alcohol will predominate, and the result will be the same whether beer, gin or whisky be taken. They are useful where great waste is going on in the system, from their power to retard the active change of tissues. If we, by the use of alcohol, can save and lengthen valuable lives, and a certain percentage of those saved fall into the habit of drinking, can they do as much harm as the other saved ones do good? It is our sacred duty to prevent, as much as in our power, the bad results of alcohol, but this does not say that we should not use it at all, as we have at present no remedy that so often compensates for the incapacity of the usual food to maintain the body in a healthy condition.

Dr. Noyes.—We don't, I think, disagree in regard to these remedies so much as it first appeared. We all agree that they are prescribed too often. This comes to pass, as Dr. McGraw has said in opening the discussion, from the too frequent habit we fall into of prescribing for symptoms, instead of trying to find out the pathology of the disease treated. Both alcohol and opium are poisons, physiologically and chemically; still more so are a great many other invaluable therapeutic agents. I believe it is a physician's business to cure disease, using to that end such remedies as in his judgment are indispensable. When his patient is relieved or cured, his duty and responsibility is discharged. The physician acts in the present, and for the present emergency; his patient, writhing in the agony of distress, or sinking into a collapse of death, must be relieved and helped out, if possible, using anything or everything known by experience to be effectual for that purpose.

Dr. Jenks.—I don't think drunkards are made by prescriptions, but entirely by conviviality. I agree perfectly with the remarks that have been made as to the use and abuse of alcohol and opium. It is a bad plan, under any circumstances, to prescribe merely for symptoms. Hunt out the pathology, give what is thought to be the best remedy, and when your patient gets well, tell him *he need not take any more medicine.*

Dr. Lathrop.—These remedies must, of course, be given with some regard to a patient's habits and propensities, but it seems to me that if we are assured in a given case that alcohol or opium is indicated, and know of no idiosyncrasy in the patient to render the medicines peculiarly dangerous, we ought to proceed and give them without hesitation.

